# Pumpkin Seed Proteins: The Potentially Alternative Protein Supplements for Food Applications

**DOI:** 10.3390/foods14223969

**Published:** 2025-11-19

**Authors:** Yufeng Xie, Yutong Wang, Xin Jin, Xinyi Zhang, Rui Yang

**Affiliations:** 1 College of Food Science and Engineering, Harbin University, Harbin 150086, China; aoaotongiswyt@163.com (Y.W.); hebxyjx@sina.com (X.J.); 2 College of Food Science and Engineering, Tianjin University of Science and Technology, Tianjin 300457, China; zhangxy011004@163.com

**Keywords:** pumpkin seed protein, plant protein, functional properties, modification, applications

## Abstract

Pumpkin seed proteins are underutilized plant proteins with a balanced content of essential amino acids and health benefits. Benefit from the functional properties and low-cost character, pumpkin seed proteins exhibit great potential for applications as alternative food ingredients. This paper reviews the composition of pumpkin seed proteins, as well as the extraction methods, including the alkaline method, ultrasonic-assisted method, and enzymatic-assisted approach. Functional properties of pumpkin seed proteins, such as solubility, foamability, gelling, and emulsification, are described. Additionally, several modification methods were emphasized to enhance the functionality of pumpkin seed proteins, including physical, chemical, enzymatic, and combined approaches. Food applications of pumpkin seed proteins, including food packaging films, meat analogs, carriers, protein supplements, and functional food additives, are also systematically introduced. Finally, the challenges and solutions that limit the widespread utilization of pumpkin seed proteins are discussed.

## 1. Introduction

Pumpkin is a common cucurbit plant primarily cultivated in Asia, Europe, and South America. According to the Food and Agriculture Organization of the United Nations, total global pumpkin production in 2021 exceeded 22.9 million tons [1]. The byproducts, including seeds and peel, are produced during the processing of pumpkin, which is frequently used to make juice and jam [2,3]. Among them, pumpkin seeds are a great source of protein, fat, fiber, minerals, phytochemicals, antioxidants, and many other nutrients. Pumpkin seeds make up 3.1% of the total weight of the pumpkin fruit and are rich in lipid (31.5–51.0%) and protein (24.0–36.5%) [4]. However, they are often processed into roasted pumpkin seeds and pumpkin seed oil in the food industry. As the production of pumpkin seed oil has increased, the output of the byproduct pumpkin seed meal has increased as well, while it is typically used in livestock feed. Over 18% of the weight of pumpkin seeds produced worldwide is removed as industrial waste every year [5]. Pumpkin seed meal contains approximately 54% protein [6]. This protein has a balanced content of essential amino acids and possesses many important biological activities, such as antioxidant, antihypertensive, antidiabetic, antibacterial, and anticancer [7]. Additionally, the in vitro digestibility of pumpkin seed meal protein is about 85.5 ± 1.7%, exceeding that of other plant proteins like sorghum (42–64%) and soybeans (75–80%) [8,9]. The predicted protein efficiency ratio (PER) of pumpkin seed protein isolate (PSPI) is 2.7, which is comparable to the protein content of unprocessed legume seeds [10,11]. As a result, pumpkin seed meal qualifies as a premium source of protein. It has great potential to be added to other food products or used as a dietary supplement.

Beyond the fundamental role in supplying amino acids for human nutrition, proteins significantly contribute to physical processes during food production, storage, and consumption [12]. Pumpkin seed proteins possess a legume-like structure, primarily composed of 12S globulin. This indicates that PSPI has comparable functional properties to legume seed proteins regarding the emulsification, foaming, and gelling properties [13]. Moreover, pumpkin seed proteins denature at temperatures slightly above 90 °C. This characteristic makes them suitable for producing specific products that necessitate the protein’s preservation in its natural form [4]. For example, PSPI retains the structural integrity and nutritional properties throughout the production process of high-temperature baked products. In the extrusion molding and high-temperature cooking procedures of plant-based meat products, pumpkin seed protein also maintains excellent texture and water-holding capacity [14,15]. However, the majority of natural proteins lack the functional properties required by food processing. Furthermore, the environment to which proteins are exposed during food processing and their interactions with macromolecules and small molecules can modify the protein structure, thereby affecting the functional properties of proteins [16,17]. Therefore, it is essential to modify the functional properties to overcome their limitations in food applications. The safety and nutritional value of pumpkin seed proteins should be considered in the selection of modification methods. Common modification methods mainly include physical, chemical, and enzymatic approaches [18].

In this paper, the composition and quality of pumpkin seed proteins are reviewed, and the extraction methods, including alkaline, ultrasonic-assisted, and enzymatic-assisted methods, are described. Along with various modification methods to enhance the properties, the functional properties of pumpkin seed proteins are highlighted, and their food applications are discussed. In addition, challenges and corresponding solutions for the applications of pumpkin seed proteins are highlighted. The aim of this review is to provide a comprehensive overview of pumpkin seed proteins and to offer useful information for their further development and application.

## 2. Composition of Pumpkin Seed Protein

### 2.1. Protein Fractions

Pumpkin seeds have garnered significant attention in recent years owing to their high protein content and functional properties as plant-based proteins. The protein content of pumpkin seeds ranges from 24.5 to 36.0 g/100 g, and this variation is influenced by factors such as growing region, variety, and climate [13,19]. In contrast, the protein content of defatted pumpkin seeds is approximately 54%. Pumpkin seed protein consists of albumin, globulin, glutelin, and prolamin. The primary storage protein in pumpkin seeds is the 12S globulin, also known as cucurbitin, which is comparable to glycinin in soybeans, legumin in peas, and cruciferin in oilseed rape. Together, the 12S globulin and 2S albumin account for 59% of total crude protein in pumpkin seeds [20]. Additionally, a small fraction of 18S globulin has been recognized as a dimeric form of the 12S protein. Cucurbitin is a hexameric protein with a molecular mass of 325 kDa, comprising six homologous subunits each measuring 54 kDa. Each subunit consists of a disulfide-linked heterodimer formed by an acidic polypeptide chain (36 kDa) and a basic polypeptide chain (24 kDa). The molecular weight of 2S albumin is approximately 12.5 kDa, which is much smaller than that of cucurbitin. The 2S albumin contains two disulfide-linked polypeptide chains with molecular weights of approximately 4.8 kDa and 7.9 kDa [21].

### 2.2. Amino Acid Composition

Amino acids serve as critical building blocks for proteins and key metabolic intermediates. Compared with the flesh and peel, pumpkin seeds have particularly high amino acid content [22]. In addition, the protein isolated from pumpkin seeds has been shown to have a high amino acid bioavailability, similar to that of soybean [23]. Notably, pumpkin seed protein contains nine essential amino acids, including histidine, which is essential for infants. Furthermore, the essential amino acid composition of pumpkin seed protein isolate basically aligns with both the FAO/WHO standards for preschool-aged children and the minimum requirements for adults (Table 1) [10].

The amino acid composition of different species of pumpkin seeds is shown in Table 1 [10,24,25,26,27,28]. Glutamic acid is the highest in pumpkin seed proteins, followed by arginine and aspartic acid, but tryptophan and cysteine are present in minimal amounts. According to the Amino Acid Score (AAS) (Table 2) [9,10], lysine is the first limiting amino acid for PSPI and Alkali fraction (AF), whereas threonine is the first limiting amino acid for Water fraction (WF) and Salt fraction (SF). As shown in Table 1, lysine is more abundant in pumpkin seed proteins, and the lysine content of *C. moschata* pumpkin seeds is 4.66 ± 0.02 g/100 g. Moreover, pumpkin seed protein contains sufficient amounts of sulfur-containing amino acids (methionine and cysteine), which are typically lacking in legume proteins.

## 3. Extraction Methods

**Figure 1:**
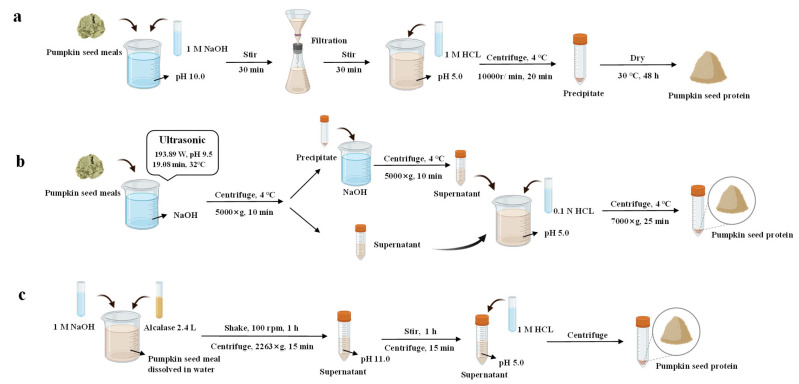
Schematic diagram of pumpkin seed protein extraction. (**a**) Alkaline extraction followed by the isoelectric precipitation method. (**b**) Ultrasonic-assisted extraction. (**c**) Enzymatic-assisted extraction.

Extraction of proteins from pumpkin seeds typically involves three primary steps. Hulled pumpkin seeds have a higher oil content and are more readily crushable than shelled pumpkin seeds. Therefore, the initial phase commences with partial dehulling and crushing the seeds. Next, most of the oil rich in unsaturated fatty acids and bioactive compounds is removed from pumpkin seeds by mechanical pressing or solvent extraction, leaving the byproducts rich in protein [28]. Finally, proteins can be extracted from the defatted pumpkin seed meal by alkaline, ultrasonic-assisted, and enzymatic-assisted methods, as illustrated in Figure 1. Additionally, Table 3 summarizes the protein content obtained through different extraction methods, along with their respective advantages and disadvantages.

### 3.1. Alkaline Extraction

The alkaline extraction method is a simple and practical technique. It not only yields proteins with a high purity but also retains the functional integrity to a certain extent, making it widely applied in the extraction of pumpkin seed proteins. This method can disrupt plant cell walls under alkaline conditions, facilitating the dissolution of intracellular proteins in aqueous solution. Meanwhile, the acidic and neutral amino acids of the proteins ionize at high pH; this effect may improve the protein extraction efficiency [35]. Temperature, pH, and extraction time are the main factors affecting the extraction efficiency. Additionally, NaCl concentration also exerts an influence on the extraction process. In particular procedures, defatted pumpkin seed meal can be generally suspended in an alkaline solution, followed by stirring to facilitate protein dissolution. The filtrate is acidified to induce protein precipitation, which can be further separated by centrifugation and dried by heating to obtain the final product (Figure 1a). This method can even achieve a protein content of 84–94% [20,29].

However, this alkaline extraction method has certain limitations. The strong alkaline environment can deteriorate some essential amino acids and induce β-elimination reactions, reducing the proportion of β-sheets, thereby adversely affecting the nutritional and functional properties [36]. Excessively high pH levels may also cause irreversible changes such as protein denaturation, amino acid racemization, and Maillard reactions, further impairing protein quality [37]. In addition to high protein content, plant protein isolates must be manufactured in a cost-effective manner to be competitive in the current market. Therefore, protein recovery rate is a crucial consideration. A study indicated that the protein recovery rate of PSPI extracted with an alkaline solution at pH 10.0 and precipitated at pH 5.0 was 36.5 ± 0.2% [20]. According to research by Achouri et al. and Rezig et al., the addition of sodium chloride can increase the protein recovery rate from defatted meal to nearly 100% [4,38]. Furthermore, this method necessitates a large amount of buffer and is time-consuming. Alternative extraction technologies, such as ultrasound-assisted extraction and enzyme-assisted extraction, have been investigated and implemented to address these limitations.

### 3.2. Ultrasonic-Assisted Extraction

As a green technology, ultrasound technology shows promising prospects and has been widely used in food processing and the extraction of bioactive compounds. This method utilizes ultrasonic energy and solvents to extract target components to produce high-value-added compounds [39]. Generally, ultrasonic-assisted extraction involves suspending defatted pumpkin seed meal in an alkaline solution, followed by ultrasonic treatment under optimized conditions. After centrifugation to collect the supernatant, the precipitate is reprocessed to maximize protein recovery. Figure 1b illustrates the ultrasonic-assisted extraction method to combine the alkali-solution and acid-isolation approach with the ultrasonic technology to obtain PSPI (Figure 1b) [30]. Ultrasonic power, ultrasonic time, and liquid-to-feed ratio have a significant impact on PSPI yield. For example, Helikh et al. achieved the highest protein yield of 81.86 g/100 g under the conditions of ultrasonic power of 456 W, ultrasonic time of 22 min, and liquid-to-solid ratio of 27 mg/L in a Box–Behnken design [31]. Another study reported that under optimized ultrasonic conditions, the resulting PSPI had a protein content of 86.07% [30]. Compared with the traditional alkaline extraction method, ultrasonic-assisted extraction offers advantages in shortening extraction time and environmental friendliness due to the cavitation effect of ultrasound.

Researchers have also examined three kinds of ultrasound-assisted extraction methods: ultrasound extraction followed by alkaline extraction (US + AE), ultrasound extraction and alkaline extraction at the same time (UAE), and alkaline extraction followed by ultrasound extraction (AE + US) [32]. All these three methods enhanced the protein yield of PSPI, with AE + US resulting in the maximum protein recovery of 57.8 ± 2.0%, the protein content of 94.04 ± 0.77 g/100 g, and the yield of 43.6 ± 0.97%. This improvement can be attributed to the frequent contact of the alkaline solution with the plant matrix and cell disruption, which facilitated the release of proteins [32]. In addition, ultrasonic treatment increased protein solubility, foaming capacity, and stability, while decreasing the water holding capacity and particle size [40]. However, excessive acoustic cavitation may cause denaturation of soluble proteins, which in turn reduces protein extraction efficiency [41]. Ultrasound-microwave synergistic extraction (UMSE) is a complementary technique that can fully exploit the high-energy effect of microwaves and the cavitation of ultrasound to overcome the limitations of both conventional ultrasound and microwave extraction [42]. The advantages of the extraction technique based on the low eutectic solvent (DES)-UMSE combination of PEG 200 are significant. It only requires a small amount of solvent and a short extraction time, yet it can achieve higher extraction yields. When the concentration of PEG 200-based DES was 28% *w*/*w*, the solid-to-liquid ratio was 28 g mL^−1^, microwave power was 140 W, and temperature was 43 °C, the protein extraction rate reached 93.95 ± 0.23%. Furthermore, this method integrates the rapidity of isoelectric point precipitation and the completeness of alcohol precipitation, and enhances the inherent volume exclusion effect in PEG 200-based DES systems [33].

### 3.3. Enzymatic-Assisted Extraction

Enzymatic pretreatment is a widely used method for facilitating protein extraction. Degradation of the cytoplasmic matrix of pumpkin seeds through enzymatic reactions can effectively release protein hydrolysates. However, the selection of proteases and hydrolysis process parameters significantly affects the extraction efficiency, functional properties, and bioactivity of the final product [43]. Tas et al. (2025) [34] proposed a method for extracting pumpkin seed protein based on Alcalase hydrolysis (Figure 1c). The pH of the pumpkin seed meal suspension was initially adjusted to 8.0, followed by the addition of 2.4 L Alcalase at 2% of the sample weight. Subsequently, after centrifugation, the supernatant was adjusted to its isoelectric point using alkaline extraction. The precipitate was recovered by centrifugation again, with a final protein extraction yield of 57.13 ± 0.65%. It is also worth noting that the extracts contained different protein fractions in previous studies, whereas only albumin was extracted in the study by Tu et al. [41]. When the catalytic activity was 5 fungal β-glucanase units per gram (FBG/g), temperature 50 °C, pH 5, and a duration of 60 min, the albumin extraction yield reached 19.1%, representing a 68% increase compared to the traditional method (11.4%) and retaining the functional properties of the albumin concentrate [41].

Comparative investigations conducted by Bučko et al. demonstrated that H1 hydrolysate produced by alcalase (89.9%) and H2 hydrolysate generated by pepsin (92.13%) exhibited greater protein purity than the original PSPI (84.87%) [29]. However, their total recoveries showed a decreasing trend, primarily attributable to the solubility limitation of pumpkin seed proteins during enzymatic digestion. Rezig et al. provided a solution to adjust process parameters that might markedly enhance protein solubility by optimizing the pH and ionic strength of the extraction process, thus enhancing recovery efficiency [4]. Future research could focus on further optimizing these parameters and exploring novel enzyme combinations to improve the efficacy of the extraction method.

## 4. Functional Properties

**Figure 2:**
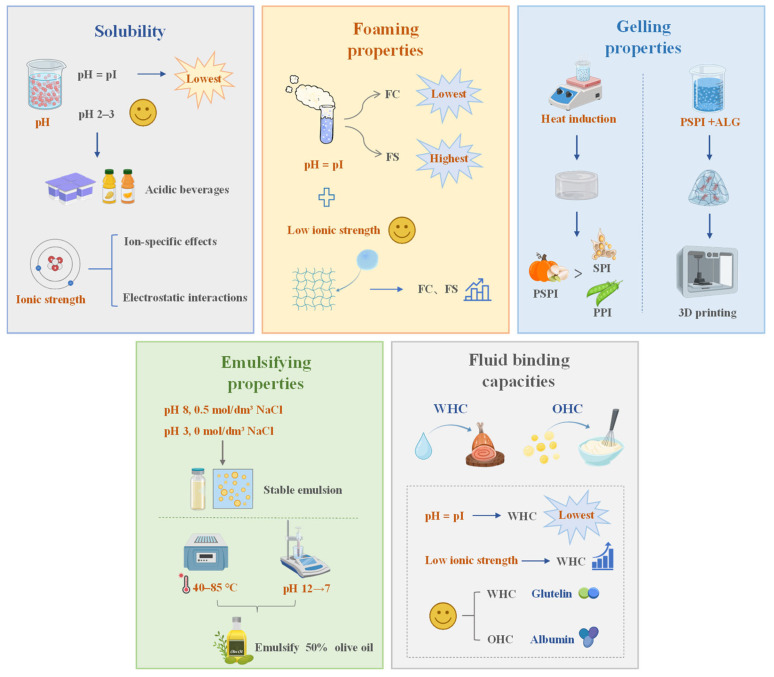
Functional properties of pumpkin seed protein.

The functional property of proteins is critical for the production, preservation, and utilization of food, as it determines the physicochemical behavior and sensory quality of final products. The properties are intrinsically associated with the protein’s physicochemical characteristics (e.g., molecular size, hydrophilicity, and hydrophobicity) and are dynamically influenced by external environmental factors, including pH, temperature, and ionic strength [44]. For instance, the solubility of PSPI reached its minimum (11%, *w*/*v*) at the isoelectric point (pH 5) due to the balanced surface charges and the enhanced hydrophobic interactions, while increasing to 68% (*w*/*v*) at pH 8, driven by the strengthened electrostatic repulsion [4]. Similarly, moderate ionic strength (0–0.2 M NaCl) improved the foaming stability of pumpkin seed albumin by 44.2%, whereas excessive salinity weakened this effect [45]. A comprehensive understanding of these properties and their regulatory mechanisms is therefore essential to tailor pumpkin seed proteins for specific food applications. A systematic analysis of the key functional properties of pumpkin seed proteins, including solubility, foaming, gelation, emulsification, and fluid holding capacity, is shown in Figure 2.

### 4.1. Solubility

Solubility is the foundational functional property of proteins, as it directly influences other properties such as foaming and emulsification. It is characterized as a thermodynamic parameter that indicates the equilibrium between protein–protein (hydrophobically driven aggregation) and protein–solvent (hydrophilically driven hydration) interactions [46]. For PSPI, solubility exhibited a strong pH dependence: the lowest solubility (11%, *w*/*v*) occurred at pH 5 (isoelectric point), where neutral surface charges promoted hydrophobic aggregation [4]. As pH deviated from 5 (either pH < 5 or pH > 5), increased surface charge density enhanced electrostatic repulsion, leading to higher solubility. The solubility of PSPI reached 68% (*w*/*v*) at pH 8 [4], and this tendency aligns with that of other plant proteins, such as sunflower and sesame proteins [38]. Notably, both the AF and PSPI exhibited substantial solubility (exceeding 30%, *w*/*v*) in the strongly acidic region (pH 2–3) [10], making them ideal functional ingredients for acidic beverages and fruit juices.

Ionic strength also modulates solubility through a combination of ion-specific effects and electrostatic interactions [47]. At pH 5 and 8, elevated ionic strength (0–0.5 M NaCl) only slightly increased PSPI solubility, indicating a limited salting-in effect [20]. In contrast, a pronounced salting-out phenomenon was observed at pH 3. Solubility diminished significantly from 38% to 13% (*w*/*v*) as NaCl concentration increased [20]. Additionally, the relationship between suspension concentration and solubility is pH dependent. Solubility of PSPI decreased with the increasing suspension concentration at pH 3 and 8, but remained unchanged at pH 5 [20]. These findings highlight that synergistic regulation of pH and ionic strength can be used to directionally modulate protein solubility by altering charge distribution and solvation state, which provides a theoretical basis for targeted functional optimization.

### 4.2. Foaming Properties

Foaming properties are critical for products like meringues and whipped creams. They are evaluated by two key indicators: foaming capacity (FC) and foaming stability (FS). FC denotes the capacity for rapid foam formation, whereas FS signifies the resistance to foam degradation [48]. Both indicators are influenced by pH, ionic strength, and protein solubility. For the three fractions of pumpkin seed protein (albumin, globulin, and glutelin), FC shows a pH-dependent behavior. Previous literature reported that the lowest FC values were observed at pH 3–4, with albumin at 35.1%, globulin at 34.9%, and glutelin at 19.3% [45]. This phenomenon is attributed to the reduced protein solubility in this pH range, which hinders the diffusion of proteins to the gas–liquid interface [49]. As pH increased to 10, FC raised significantly; the corresponding values were 109.7% for albumin, 69.3% for globulin, and 49.1% for glutelin [45]. The augmentation is propelled by the improved net charge, the diminished hydrophobicity, and the increased structural flexibility of proteins [18]. All these elements facilitated swift protein reorganization and bubble encapsulation at the interface.

In contrast, FS is optimal for all protein fractions near their isoelectric points. At neutral surface charge, the weakened intermolecular electrostatic repulsion facilitates the formation of a cohesive viscoelastic interfacial film, thereby improving foam stability [45]. Supporting this, Du et al. further demonstrated the systematic improvement of the FC and FS indexes through the modulation of the pH environment [50]. Ionic strength additionally influenced foaming properties: within the range of 0–0.2 M NaCl, FC increased by 67.9% (albumin), 25.3% (globulin), and 61.8% (glutelin), while FS rose by 44.2% (albumin), 33.2% (globulin), and 33.1% (glutelin) [45]. This phenomenon results from the increased protein solubility at a low ionic strength, facilitating the development of stable protein films encasing foam bubbles, while excessive salinity (>0.2 M NaCl) reduced both FC and FS by disrupting interfacial film integrity.

### 4.3. Gelling Properties

Protein gels regulate the microstructure, texture, and stability of foods such as meat analogs and yogurts. This makes gelation a key property for texture-focused applications [51]. Proteins derived from pumpkin seeds possess intrinsic gel-forming capabilities. The hydrophobic amino acid content of PSPI is 35.67 mol%, which significantly exceeds the 32.5 mol% threshold required for coagulation gel formation [52]. The gel properties of the three pumpkin seed protein fractions vary with pH. This variation is reflected by the least gelation concentration (LGC), defined as the minimum protein concentration needed for gel formation. Albumin exhibited the lowest LGC of 12% at pH 3–4 [45]. The minimum LGC values for globulin and glutelin were 14% and 12%, respectively, recorded at pH 4–6 [45]. Under extreme pH conditions (pH 2 and 10), all three fractions showed higher LGC. The elevated net charge intensifies intermolecular electrostatic repulsion, hence obstructing gel network development [45].

Salt ions further modulate gel behavior through charge shielding. Increasing NaCl concentration from 0 to 0.2 M reduced the LGC of all three fractions, promoting protein aggregation [45]. However, at higher salt concentrations (>0.2 M), albumin’s gelation capacity decreased, while globulin and glutelin continued to improve. This divergence is probably attributable to variations in conformational reactions to salt [45,53]. Heat-induced gelation is particularly relevant for food processing. Native PSPI exhibited superior heat-induced gel properties compared to soy protein isolate (SPI) and pea protein isolate (PPI) [52]. After ultrasonic or alkaline treatment, the gel strength and storage modulus (G’) of these three plant proteins increased, with PSPI exhibiting the most favorable results. This improvement is attributed to increased soluble aggregates [54]. In contrast, acid treatment formed insoluble aggregates of PSPI and SPI, which reduced solubility and gel quality [52].

To address the restricted gelling ability of PSPI, the incorporation of alginate (ALG) with PSPI has been proposed [55]. At low ALG concentrations (2–3%), the PSPI-ALG hydrogel demonstrated textural qualities such as hardness and elasticity similar to gelatin. At concentrations greater than 4%, hardness increased significantly while elasticity and cohesiveness remained unchanged [55]. Importantly, the PSPI-ALG hydrogel had superior thermal stability and maintained structure at 90 °C compared to gelatin. The PSPI-ALG hydrogel is a cost-effective and sustainable substitute for gelatin, exhibiting improved stability and thus being appropriate for food 3D printing. This provides insights for developing functional plant-based alternatives to animal gelatin.

### 4.4. Emulsifying Properties

Proteins act as natural emulsifiers by reducing oil–water interfacial tension and preventing droplet aggregation. Emulsifying properties are evaluated by emulsification activity (EA, ability to form emulsions) and emulsification stability (ES, resistance to droplet aggregation) [56]. PSPI exhibited adsorption capacity at both gas/liquid and oil/liquid interfaces, with particularly stable emulsions formed at pH 8 (ionic strength 0.5 mol/dm^3^ NaCl) and pH 3 (ionic strength 0 mol/dm^3^ NaCl) [20]. Furthermore, when a PSPI solution at pH 12 was subjected to gradient heating (40–85 °C) and subsequently adjusted to pH 7, the size of the emulsion droplets exhibited a systematic decrease with the increase in treatment temperature. All protein variants demonstrated the capability to emulsify up to 50% of olive oil [57]. This phenomenon is attributed to heat-induced unfolding of the protein structure, which enhances adsorption efficiency at the oil droplet interface and consequently boosts interfacial stability.

Comparative studies with SPI showed that PSPI ranked second in emulsion activity. The value of PSPI was 0.930 ± 0.015 while that of SPI was 0.990 ± 0.001 [10]. Regarding ES, the WF (30.09 ± 0.38) demonstrated superior stability compared to SPI (27.41 ± 1.17), followed by PSPI (23.65 ± 3.15) and AF (14.95 ± 0.26). Additionally, the WF emulsion exhibited a consistent and homogeneous dispersion in the absence of salt. With increasing salt concentration, flocculation occurred, but no significant aggregation was observed [10]. This phenomenon can be ascribed to its excellent interfacial properties. Low ionic strength promotes emulsion stability owing to the electrostatic repulsion between oil droplets [58]. In conclusion, PSPI demonstrates excellent emulsifying properties comparable to SPI, with superior stability in certain conditions, making it a promising plant-based alternative for food emulsion applications.

### 4.5. Fluid Binding Capacities

Fluid binding capacities include water holding capacity (WHC) and oil holding capacity (OHC). WHC refers to the ability to retain water, while OHC refers to the ability to retain oil. These capacities affect the texture, juiciness, and shelf life of foods [59]. The WHC of PSPI was 1.2–1.35 mL/g while the alkaline fraction exhibited a WHC of 1.48 to 1.50 mL/g. The OHC of PSPI ranged from 3.59 to 3.70 mL/g, marginally lower than that of SPI, which ranged from 3.78 to 3.98 mL/g, although surpassing cowpea protein at 1.10 mL/g, sesame seed protein at 1.5 mL/g, and ginkgo seed protein at 2.95 mL/g [10]. Notably, the OHC of the alkaline fraction was 2.95 mL/g, which was comparable to that of ginkgo seed protein.

The pH and ionic strength influence the fluid binding capacities of albumin, globulin, and glutelin. The WHC was minimized near the isoelectric point, which occurred at pH 4–5 for all three fractions. As the pH shifted to 2 or 10, the WHC increased [45]. Low ionic strength, specifically in the range of 0–0.2 M (NaCl), enhanced the WHC by 20.9%, whereas excessive salinity diminished this impact. Among the fractions, glutelin consistently exhibited higher WHC than globulin, whereas albumin had the lowest values. In contrast, compared to globulin and glutelin, albumin had the highest OHC of 6.90 mL/g [45]. These differences in fluid binding capacities enabled targeted selection of pumpkin seed protein fractions for specific applications. For example, glutelin could be utilized for moisture-rich products such as processed meats, and albumin could be used for oil-based products such as bakery batters.

## 5. Modification Methods

Due to inappropriate functionality, the utilization of pumpkin seed proteins may be restricted. For example, the isoelectric point of pumpkin seed proteins falls within the acidic range, rendering them insoluble in acidic circumstances, hence limiting their application in acidic foods. In addition, the protein extraction process has a negative impact on the natural structure of the proteins, affecting the solubility and thus the ability to be incorporated into the food system. Therefore, different modification techniques need to be investigated to enhance the functionality of pumpkin seed proteins (Table 4).

### 5.1. Enzymatic Modification

Enzymatic modification is a sustainable and efficient approach for functionalizing pumpkin seed proteins. One can use specific proteases to hydrolyze peptide bonds, to transform native proteins into bioactive peptides or functional amino acid sequences. This method offers advantages like environmental compatibility and energy efficiency without toxic residues [80]. The specific protease and hydrolysis parameters (temperature, pH, and enzyme-to-substrate ratio) significantly influence the modification effect. Within the pH range of 5–10, the solubility of the hydrolysates consistently exceeded 55%. Notably, the hydrolysate generated with neutral protease had the maximum solubility at 86.24 ± 2.92%. The specific protease markedly affected the surface hydrophobicity and thermal properties of the pumpkin seed protein hydrolysates [61]. Furthermore, enzymatic treatment increased the content of antioxidant amino acids. At a concentration of 0.2 mg/mL, the hydrolysate generated with acidic protease exhibited the highest ABTS^+^ radical scavenging rate of 72.22 ± 1.43% [61]. The incorporation of enzymatically hydrolyzed PSPI into food formulations provides superior functional properties compared to the native protein.

Alkaline protease and pepsin were used to hydrolyze the protein separately, yielding hydrolysates H1 and H2, both of which showed significantly improved solubility near their respective isoelectric points [20]. Both hydrolysates effectively preserved emulsion stability over diverse pH levels and ionic strengths, demonstrating superior stability in surface pressure properties relative to the native proteins [20]. This might have been due to the fact that enzymatic hydrolysis decomposed proteins into small-molecular-weight peptide fragments, which could more easily expose both hydrophobic and hydrophilic residues within the same molecular structure. During emulsion formation, the hydrophobic side chains of the peptide fragments could interact with oil droplets to achieve oil-phase adsorption. The alkaline protease hydrolysate exhibited superior interfacial activity under isoelectric point conditions [60]. Furthermore, the interfacial film formed by its adsorbed layer demonstrated a higher elastic modulus (E’) than that of PSPI. At pH 3, PSPI demonstrated a gradual decline in E’ as ionic strength increased (0–500 mM NaCl). In contrast, its enzymatic hydrolysates demonstrated a significant increase in E’ under the identical conditions, indicating an enhanced ionic tolerance of the modified peptides. Kinetic analyses revealed that both the diffusion rate constant and the adsorption rate constant of the hydrolysate exceeded those of PSPI [60].

Mazloomi-Kiyapey et al. found that pepsin hydrolysis of pumpkin seed powder produced hydrolysed products with an enhanced emulsifying capacity compared to native proteins across the pH range of 4–10, achieving optimal performance at pH 10 [62]. Meanwhile, the OHC of the enzymatic hydrolyzed product was enhanced, which was directly associated with the elevated encapsulation rate of oil droplets and the increased surface hydrophobicity. Under optimal reaction conditions (enzyme-to-pumpkin seed protein isolate mass ratio of 1:100, reaction time of 12 h), the solubility of pumpkin seed protein isolate increased from 19.35% to 44.73%, while its foaming capacity significantly improved from 23.33% to 80% [63]. This performance enhancement could be attributed to protein-glutaminase-mediated deamidation, a process that altered the net charge distribution, molecular structure, and conformation of pumpkin seed proteins. Such modifications not only enhanced the solubility of the proteins themselves but also promoted the diffusion and adsorption of large amounts of pumpkin seed proteins at the gas–liquid interface [63]. These enhancements make deamidated PSPI appropriate for high-solubility applications and emulsifier-based products, such as protein shakes and plant-based cheese. Furthermore, the slight increase in glutamic acid and arginine content, along with the preservation of the protein backbone, contributes to the nutritional value of deamidated pumpkin seed protein.

Enzymatic protein hydrolysis facilitates the precise generation of bioactive peptides by selectively cleaving peptide bonds inside proteins. The specificity of proteases dictates the terminal amino acid sequences of the released peptides. Peptides containing hydrophobic amino acids at the C-terminus can act as potent angiotensin-converting enzyme (ACE) inhibitors by competitively binding to the enzyme’s active site, while those with electron-donating residues such as tyrosine and tryptophan can stabilize free radicals and confer antioxidant activity [81]. When the degree of hydrolysis (DH) reached 53.23%, bioactive peptides were acquired, with a high ACE inhibitory activity. These peptides had a molecular weight of <15 kDa and a half-maximal inhibitory concentration (IC_50_) of 0.422 mg/mL, whereas the native protein exhibited no such activity [82]. Under identical conditions, alkaline proteases exhibited enhanced efficacy compared to flavored proteases. The hydrolysates produced by alkaline proteases showed a free radical scavenging activity of 7.59 ± 0.081 mM TEAC/mg within 60 min [64]. These hydrolysates further exhibited promising pH stability and thermal stability, indicating their suitability as natural antioxidants.

Temperature optimization study indicated that Aspergillus niger pepsin I generates antioxidant-rich hydrolysates most effectively at approximately 50 °C [65]. Compared with the alkaline protease hydrolysate, the trypsin hydrolysate exhibited enhanced DPPH radical scavenging capacity, total antioxidant activity, and ferric ion chelating capacity. These characteristics promoted its application potential as a natural antioxidant. The disparity in antioxidant activity between the two hydrolysates resulted from their differences in the length and structure of the hydrolyzed peptides. As a specific endoprotease, trypsin could more easily cleave peptide bonds at specific sites within the peptide chain and release short chains rich in hydrophobic amino acids [25]. Advancements have been achieved in the utilization of innovative proteases for the digestion of pumpkin seed protein. For instance, arginine aminopeptidase (BAAP) derived from the *Bacillus axarquiensis* SWJSX8 strain has been shown to increase the protein hydrolysis rate of pumpkin seeds by 5.7–8.2%. Beyond boosting hydrolysis efficiency, this enzyme also elevated the small peptide content of the hydrolysate and enhanced its antioxidant activity [66]. Collectively, enzyme modification technologies demonstrate environmentally sustainable and efficient approaches to optimizing the functional properties of PSPI. Through targeted hydrolysis, these methods not only improve key functional properties, including solubility, interfacial activity, and emulsion stability, but also release bioactive peptides with valuable biological effects, such as ACE inhibitory activity and antioxidant capacity. These findings provide a robust foundation for future innovations in food technology, particularly in the development of health-promoting functional foods and nutraceuticals.

### 5.2. Physical Modification

#### 5.2.1. Conventional Thermal Technology

Heating provides activation energy to disrupt peptide bonds, leading to the disruption of protein tertiary structure. Heating can transform the protein from native conformation to semi-molten spheres with enhanced functionality [83]. The FC of the raw pumpkin seed powder sample was 75.6%, which was enhanced to 83.0%, 80.4% and 78.8% by conventional heat treatment, microwave, and ultrasonic treatments, respectively. Among them, the conventional heat treatment exhibited the most significant FC enhancement [9]. In addition, the WHC of pumpkin seed protein was improved by 22% from 1.55 g H_2_O/g to 1.90 g H_2_O/g after thermal treatment. Similarly, after heat treatment, the OHC increased from the initial 0.78 g oil/g protein to 0.90 g oil/g protein, equivalent to the OHC of samples subjected to microwave and ultrasound treatments [9]. Lovatto et al. (2020) also obtained similar oil-holding capacity results (0.72 g oil/g) in their study on phosphorylated protein concentrates from pumpkin seed meal [72]. These results confirmed that pumpkin seed proteins, as a plant protein with high water-holding and oil-holding capacities, possess extensive potential applications in meat products and meat alternatives.

#### 5.2.2. Non-Thermal Technology

Non-thermal methods such as ultrasound and high-pressure homogenization (HPH) are progressively employed for pumpkin seed protein modification due to their safety, environmental friendliness, and sustainability [84]. High-intensity ultrasound (HIU) modifies the secondary and tertiary structures of PSPI through cavitation effects, enhancing solubility, emulsification, and foaming properties [50]. At identical pH levels, the FC of treated proteins was double that of the control group. Notably, a mere 5 min of treatment was adequate to improve FS and emulsification characteristics [67]. Interestingly, ultrasonicated PSPI suspensions at pH 2.5–6.5 exhibited both the highest emulsifying activity and the lowest emulsion instability index [30], a characteristic that proves particularly valuable for food processing applications such as beverage and yogurt production. Furthermore, compared to the untreated sample (0.90 ± 0.08 g/g), ultrasonicated PSPI exhibited an OHC of 1.20 ± 0.07 g/g, representing a 33% increase [30]. Meanwhile, this technology improved the color characteristics of PSPI. After HIU treatment, its lightness value and yellowness value increased. This change might have been attributed to the structural modification of pigments caused by the cavitation effect during ultrasound treatment, thus increasing the commercial value for the food sector by improving the visual appeal of products. It is noteworthy that when ultrasound was applied as a pretreatment or during the hydrolysis process to alkaline protease and pumpkin seed protein concentrate (substrate), the rate of protein hydrolysis can be significantly accelerated [68]. Compared to the hydrolysates obtained through conventional hydrolysis methods, the resulting hydrolysates showed superior concentration, solubility, and free radical scavenging capacity.

HPH utilizes high turbulence and hydrodynamic shear (up to 200 MPa) to disrupt the tertiary and quaternary structures of proteins through hydrogen bond cleavage, thereby influencing the functional properties of proteins [69,85]. Following the combined application of 100 MPa HPH with AE, the solubility of PSPI increased to 30.21 ± 0.93%, indicating a 1.4-fold improvement relative to untreated samples. This improvement could be attributed to the disruption of tertiary and quaternary protein structures through hydrogen bond cleavage, coupled with increased surface hydrophobicity resulting from HPH treatment [86]. Furthermore, HPH effectively improved the emulsification index of PSPI by inducing protein unfolding and increasing surface activity, regardless of whether AE was used as a pretreatment or post–treatment method [69]. Among these, the optimal emulsification performance was observed when AE was followed by HPH at 50 MPa, with an emulsification index of 51.14 ± 1.16%. In contrast, the maximum foam formation occurred when AE was paired with HPH at 100 MPa, reaching 119.17 ± 9.17%. While HPH treatment increased the FC to 24.58 ± 5.57%, it concurrently led to a decrease in FS [69]. These findings systematically reveal the mechanism by which ultrasound and HPH technologies influence protein functionality by modulating protein conformational changes, and also offer a theoretical basis for the functional improvement of plant proteins.

### 5.3. Chemical Modification

Chemical modification improves protein functionality through reactions between proteins and chemical reagents (e.g., acetylation and phosphorylation). It offers advantages like strong specificity and high efficiency, but requires attention to safety and nutrient retention [80]. pH-shifting treatment represents a promising approach for modifying the thermal, structural, and functional properties of PSPI. Under controlled heating conditions, PSPI exhibits denaturation temperatures of 87.67 °C and 104.11 °C [87]. Notably, compared with the control group, the PSPI samples treated only at pH 2 showed no significant differences in denaturation temperature or enthalpy, which indicated that specific pH conditions could effectively maintain the thermal stability of the protein. Among these treatments, pH 2, 4, and 12 could enhance the emulsifying properties of PSPI [70], and this phenomenon might be attributed to the synergistic effect of high disulfide bond content, low free sulfhydryl groups, and small-sized soluble proteins. Further study found that extreme pH treatments (pH 1.5 or 12.0) had a more significant effect on improving protein surface activity and emulsion stability [70]. At the same time, treatment at pH 12.0 could increase the solubility of PSPI from 11.5% to 38.3%, and simultaneously improve its antioxidant activity, WHC, OHC, and foaming properties [18]. This broadened the potential application scenarios of pumpkin seed protein in the field of food processing.

Acylation using acetic anhydride is one method employed to modify the functional properties of pumpkin seed protein [71]. Even at a dosage of 0.4 mL/g, acetylation significantly enhanced the WHC, OHC, and emulsifying properties of pumpkin seed protein concentrate. Furthermore, in comparison to natural pumpkin seed protein, the foaming properties of the acetylated products (with 2.0 mL/g acetic anhydride) were enhanced. Phosphorylation modification, using sodium trimetaphosphate under optimized conditions (concentration 4.00%, pH 4.5), yielded the highest protein extraction rate. The modified product also demonstrated reduced levels of anti-nutritional factors and fiber content, alongside significantly improved protein digestibility and essential amino acid content [72]. These enhancements expand their potential applications in the food industry. In addition, protein fibrils generated via the self-assembly of structured cross-β-sheets have attracted considerable interest owing to their distinctive functional attributes. Protein fibrillation under acidic heating (pH 2.0, 85 °C, 0–72 h) formed ordered cross-β-sheets, and PSPI contained abundant hydrophobic fibril-forming regions, with 11S globulin contributing 32 unique peptides and 2S albumin contributing 12 unique peptides [73]. This study provides valuable insights into the fibrillation kinetics of PSPI and lays the foundation for its future development in innovative foods and applications as functional biocompatible materials.

Bioactive components also affect the physicochemical properties and functions of proteins. Yang et al. found that under the condition of pH 9.0, PSPI formed a complex with a higher relative molecular mass and a more stable secondary structure after covalent coupling with polyphenols [74]. This phenomenon can be attributed to the fact that the alkaline environment can moderately unfold the protein conformation by promoting an increase in the content of α-helix and β-sheet structures, thus enhancing the overall structural stability of the protein [74]. When PSPI underwent a high degree of covalent coupling with pyrogallic acid (1,2,3-benzenetriol) in different proportions, more hydrophobic groups in the protein molecules were exposed, further strengthening its antioxidant activity [13]. At the same time, the cross-linked network formed by covalent bonds could restrict the thermal movement and conformational unfolding of protein molecules in high-temperature environments, effectively delaying the thermal denaturation process [13]. Compared to PSPI, the covalent complex of PSPI and epigallocatechin-3-gallate (EGCG) exhibited a similar endothermic peak but an elevated denaturation temperature, with a peak denaturation temperature reaching 83.15 °C [75]. Furthermore, the conjugation of EGCG to PSPI led to substantial alterations to its functional properties. The solubility of the PSPI-EGCG covalent complex increased by 66.07%, its maximum emulsifying activity reached 421.91 m^2^/g, and it demonstrated radical scavenging activities of 82.73% against DPPH and 90.70% against ABTS [75]. Current studies have clarified the positive effects of the interaction between polyphenols and PSPI; however, existing research predominantly focuses on in vitro single systems, neglecting the potential interference of other components in the food matrix, such as carbohydrates and lipids. Therefore, the stability and functional retention rate of such complexes during actual food processing still need to be further verified through experiments.

### 5.4. Combined Modification

In certain instances, using only one method to modify natural proteins may be insufficient to overcome their functional defects. To minimize the adverse effects of heat treatment and reduce toxic by-products generated by chemical modification, researchers have investigated combined modification approaches to address these deficiencies and synergistically improve the functionality of proteins [88]. For example, the pH-HIU coupling system demonstrated the strongest synergistic effect among pH alteration, HIU, and high-pressure processing (HPP) applied individually [18]. The modified protein particles demonstrated a more uniform particle size distribution, elevated solubility (69.1%), and enhanced WHC, OHC, and foaming properties. Concurrently, the EAI and ESI reached 43.65 m^2^/g and 38.4 min, respectively. Furthermore, pH modification combined with HIU improved thermal stability and protein digestibility while reducing viscosity, thereby significantly enhancing structural and functional properties. Another sample is that the heat-assisted pH shifting (HP) treated-PSPI exhibited promise as a high-performance food-grade Pickering emulsifier, with an internal phase volume ratio of 80% (*v*/*v*) [57]. This emulsion remained stable under centrifugation at 10,000× *g* for 60 min, highlighting its suitability for industrial applications.

The enzymatic hydrolysis method has advantages, such as sustainability, but it also suffers from high enzymatic hydrolysis costs and long hydrolysis cycles [89]. To address these limitations, ultrasonic and microwave treatments were combined with enzymatic digestion to enhance catalytic efficiency [77]. Pre-ultrasonic treatment and ultrasonic-assisted hydrolysis could increase the protein hydrolysis rate by 280% and the soluble peptide concentration by 24%. Mild pre-ultrasonic treatment can increase enzyme activity, thereby enhancing the hydrolysis rate and degree of pumpkin seed protein hydrolysates. This process can increase protein solubility by 53% and increase in vitro antioxidant activity by 40% [77]. The application of ultrasound could also enhance the catalytic efficiency of Brauzyn enzyme, Flavourzyme, and neutral enzyme [78], and the resulting hydrolysates exhibited both better antioxidant performance and higher solubility over a wider pH range. Similarly, microwave pretreatment (500–900 W for 30–90 s) combined with trypsin digestion generated highly bioactive products, achieving 95.5% iron chelation activity and 51.5% DPPH radical scavenging capacity at an enzyme–substrate ratio of 1.5% [79]. These improvements render the combined utilization of different technologies suitable for applications in the food industry.

## 6. Biological Activities

Pumpkin seed protein possesses various biological activities. When proteins are hydrolyzed, the peptides with enhanced biological activity and health-promoting effects can be obtained, which also hold potential industrial application prospects [90]. As aforementioned, the enzymatic hydrolysis of pumpkin seed protein improves its functional properties and generates peptides with potential biological characteristics. The pumpkin seed protein and peptides offer a variety of health benefits, such as antioxidant, antihypertensive, antidiabetic, antibacterial, and anticancer activities.

### 6.1. Antioxidant Activity

Many studies on the biological properties of hydrolyzed pumpkin seed protein have focused on antioxidant activity. The antioxidant activity of pumpkin seed protein hydrolysates is commonly evaluated using various in vitro assays, including DPPH free radical scavenging capacity assay, oxygen radical absorbance capacity (ORAC) assay, total antioxidant capacity assay, and ferrous ion chelating capacity assay [91]. The efficacy of the antioxidant action is intricately linked to the amino acid content, peptide molecular weight, and structural characteristics of the hydrolysates. Among these, the content of hydrophobic amino acids (such as phenylalanine, tyrosine, and leucine) is a key influencing factor. These amino acids can scavenge free radicals through hydrogen donation and terminate oxidative chain reactions [62]. Meanwhile, peptides with smaller molecular weights generally exhibit stronger antioxidant activity. For example, most peptides with a molecular weight of less than 6.5 kDa obtained by Alcalase hydrolysis showed potent antioxidant capacity [25]. In addition, covalent coupling of polyphenols with proteins could augment the antioxidant efficacy of pumpkin seed protein by incorporating hydroxyl groups, hence enhancing its molecular antioxidant activity [13].

The antioxidant activity of pumpkin seed protein hydrolysates is affected by multiple factors. The enzyme employed for hydrolysis and its selectivity significantly influence the activity. For instance, the total antioxidant activity and metal ion chelating activity of Alcalase hydrolysates were higher than those of trypsin hydrolysates, while chymotrypsin hydrolysates exhibited a higher ORAC value [92]. The association between DH and antioxidant activity is not merely a straightforward positive connection. Research indicated that an extremely high level of hydrolysis resulted in diminished antioxidant activity, while a lower level of hydrolysis was more favorable for producing high-activity hydrolysates [69]. Hydrolysis temperature also plays a pivotal role. At a temperature of approximately 50 °C, protein molecules unfolded to expose internal hydrogen-donating residues, thereby enhancing the antioxidant activity of the hydrolysates [65]. Furthermore, the pretreatment method, purification process, and subsequent embedding technology of pumpkin seed protein can also affect the antioxidant stability. For example, embedding with sodium carboxymethylcellulose–alginate composite hydrogel effectively slowed the decline in antioxidant activity of pumpkin seed protein hydrolysates during simulated gastrointestinal digestion [93]. Through the rational selection of enzymatic hydrolysis systems and optimization of process parameters, hydrolysates and peptides with excellent antioxidant properties can be efficiently prepared from pumpkin seed protein, providing an effective approach for the development of natural antioxidants and functional foods. Nevertheless, existing studies on the in vivo antioxidant mechanisms of pumpkin seed protein hydrolysates remain inadequate, necessitating further comprehensive investigation to confirm their stability, safety, and efficacy in complex food systems.

### 6.2. Antihypertensive and Antidiabetic Activities

Hypertension, a principal risk factor for global cardiovascular illnesses, results in numerous fatalities annually. Nonetheless, clinically used synthetic antihypertensive drugs are often accompanied by many side effects [94]. Therefore, the development of natural-source bioactive components with antihypertensive effects has become a research focus in the fields of nutrition and medicine. As a highly promising natural protein resource, the pumpkin seed protein hydrolysates have been confirmed to have antihypertensive activity. Pumpkin seed protein hydrolysates are reported to have ACE inhibitory activity, which is a key indicator for evaluating antihypertensive potential [95]. For example, utilizing alkaline protease to hydrolyze pumpkin oil cake protein resulted in a degree of hydrolysis of 53.23 ± 0.7%, yielding hydrolysates with an ACE inhibition rate of 71.05 ± 7.5% (IC_50_ = 0.422 mg/mL) [64]. After gastrointestinal digestion of transglutaminase cross-linked modified pumpkin oil cake protein, the ACE inhibitory activity of its hydrolysates (IC_50_ = 0.28 ± 0.01 mg/mL) was comparable to that of natural pumpkin seed protein hydrolysates (IC_50_ = 0.30 ± 0.04 mg/mL), and the cross-linking treatment did not affect the digestive properties and bioactivity [96]. A new tripeptide, IAF (Ile-Ala-Phe), was discovered from pumpkin seed protein using computer screening methods [76]. This peptide exhibited favorable bioavailability, with an IC_50_ value for ACE inhibitory action of 19.87 ± 0.50. Molecular dynamics simulations confirmed that IAF stabilized the complex by forming hydrogen bonds with key residues His513 and Glu162 in the active site of ACE, as well as chelation between the O3 atom and Zn^2+^, thereby exerting a potent inhibitory effect [76]. In addition, functional biscuits including pumpkin seed components demonstrated considerable ACE inhibitory activity following in vitro digestion [97], indicating that bioactive peptides derived from pumpkin seed protein can maintain antihypertensive potential in practical food applications.

Diabetes mellitus, a global chronic metabolic disease characterized by persistent hyperglycemia, has a rising prevalence rate. The development of safe and effective natural hypoglycemic components is of great significance [98]. Pumpkin seed protein and its hydrolysates exhibit significant potential in combating diabetes, mostly by reducing the activities of α-amylase and α-glucosidase, which delay starch breakdown and glucose absorption. Biscuits made from pumpkin pulp, pumpkin seeds, and pumpkin starch residue had inhibition rates of 59% and 69% against α-amylase and α-glucosidase, respectively, and their glycemic index (GI) was only 50–52%, belonging to low-GI foods, which was significantly lower than that of wheat flour biscuits (GI = 61.43%) [15]. Adding 20% or 35% pumpkin seed press cake powder into gluten-free biscuits resulted in a substantial reduction in the predicted glycemic index of the products, decreasing from 67.8 ± 0.3 in the control group to approximately 60. Furthermore, when pumpkin seed protein synergizes with active components of beetroot juice, it can further enhance its α-glucosidase inhibitory activity. Among them, the digestive juice of biscuits added with 20% microcapsules showed the strongest inhibitory ability (IC_50_ = 0.53 mg) [99]. These results indicate that pumpkin seed protein can not only produce direct hypoglycemic bioactive peptides through enzymatic hydrolysis but also exert potential antidiabetic effects by regulating the nutritional composition and digestive properties of products in food matrices. Although pumpkin seed protein hydrolysates have demonstrated notable antihypertensive and hypoglycemic effects in in vitro studies, existing research predominantly remains at the in vitro level, lacking systematic animal model verification and human clinical trial data.

### 6.3. Antibacterial and Anticancer Activities

Natural antibacterial components and plant extracts have attracted extensive attention from scientists in the fields of food and medicine due to their potential application value in the food and pharmaceutical industries. Crude protein extracts from pumpkin seeds exhibit high antibacterial activity against Gram-positive bacteria such as *Staphylococcus aureus* and *Bacillus subtilis*, with the diameters of the inhibition zones reaching 10.0 mm and 8.0 mm, respectively, while exhibiting no notable inhibitory effect on Gram-negative bacteria [100]. In addition, specific proteins in pumpkin seeds, such as three basic proteins (MAP2, MAP4, and MAP11), can inhibit the growth of yeasts. Pepocin, an antifungal peptide derived from black pumpkin seeds, also exerts a significant inhibitory effect on fungi, including *Botrytis cinerea*, *Fusarium oxysporum*, and *Mycosphaerella lavendulae*. However, γ-irradiation led to a decrease in the antibacterial activity of pumpkin seed protein as the irradiation dose increased, which may be related to protein cross-linking or aggregation induced by radiation [101]. Further comprehensive investigations are required to validate and enhance the utilization of pumpkin seed protein in food preservation and pharmaceuticals.

Cancer is one of the leading causes of death worldwide, and its incidence is continuously rising, especially in low-income countries. The consumption of nutritious, natural foods abundant in bioactive compounds holds significant potential for diminishing cancer risk and incidence [102]. The 2S albumin from pumpkin seeds has been proven to possess strong anticancer activity, exerting significant cytotoxic effects on various cancer cell lines such as breast cancer (MCF-7), ovarian teratocarcinoma (PA-1), prostate cancer (PC-3 and DU-145), and hepatocellular carcinoma (HepG2) [103]. After treatment with 20 μmol/L pumpkin 2S albumin, the survival rates of these cancer cell lines were significantly reduced, among which MCF-7 cells were the most sensitive, and their cell viability could be significantly inhibited at a concentration of 5 μmol/L. Subsequent research indicates that the anticancer mechanism of pumpkin 2S albumin was mediated by the induction of apoptosis in cancer cells, as evidenced by acridine orange–ethidium bromide staining and DNA fragmentation analysis, with its apoptotic effect being dose-dependent. In addition, pumpkin 2S albumin also has DNase activity, which can hydrolyze the DNA of cancer cells, and this may also provide certain support for its anticancer activity [103]. However, current studies on the anticancer effects of pumpkin seed protein and its hydrolysates in animal models and humans are still relatively limited. In the future, more in vitro and in vivo experiments need to be carried out to further explore their anticancer mechanisms and potential clinical application value.

## 7. Food Applications

**Figure 3:**
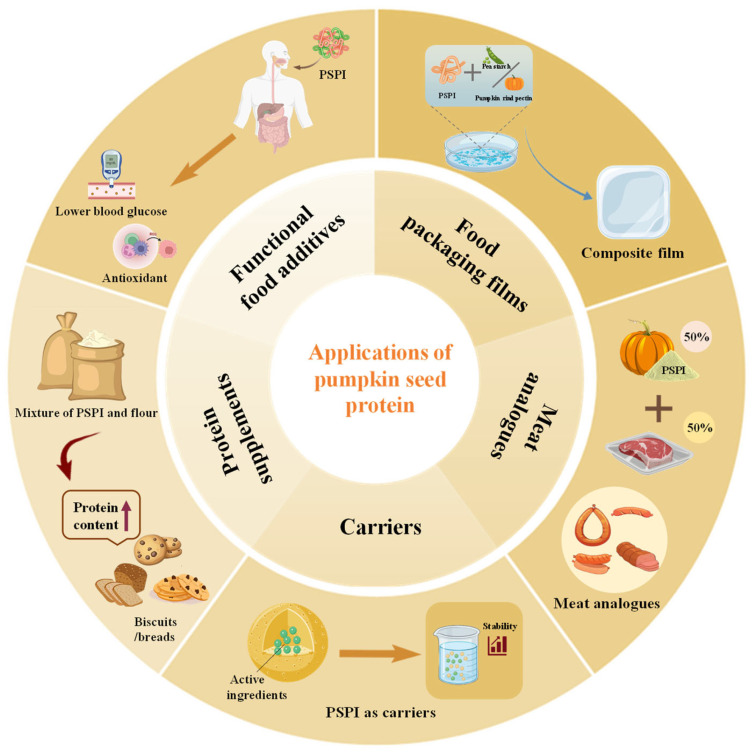
Applications of pumpkin seed protein in the food industry, including food packaging films, meat analogs, carriers, protein supplements, and functional food additives.

In recent years, pumpkin seed proteins and their derivatives have gained significant attention for their application in the food industry, primarily due to their superior nutritional composition and functional properties. In addition, utilizing agro-industrial production losses to recover proteins may serve as a sustainable strategy to enhance the economic viability of these byproducts. Pumpkin seed protein demonstrates significant potential for diverse food applications, as illustrated in Figure 3, including food packaging films, meat analogs, carriers, protein supplements, and functional food additives.

### 7.1. Food Packaging Films

Pumpkin seed protein films are elastic and have the advantage of blocking gases. However, their maximum tensile strength was only 6 MPa, which was inferior to that of gelatin cross-linked and corn protein films [104,105]. Moreover, the inherent hydrophilicity of the protein greatly restricts its practical application [105]. In high-humidity environments or upon direct exposure to moisture, the mechanical strength and barrier characteristics of protein films diminish due to water absorption. To address these issues, several studies have explored the development of pumpkin seed protein-based food packaging films. For instance, PSPI can be used to produce biodegradable films with different pH values and plasticizer dosages [105]. Films formed at all pH values except in the 4–8 range. When glycerol was added at 0.4 g/g PSPI, the films exhibited excellent barrier properties against O_2_, N_2_, and CO_2_. Compared to pure PSPI films, these biodegradable films demonstrated a higher elongation at break and permeability, making them suitable as air barrier stretch coatings [105].

To further enhance the film properties, researchers attempted to composite pumpkin seed protein with other substances. Lalnunthari et al. [6] first reported a composite film system composed of pumpkin seed protein and pumpkin peel. The film, composed of defatted pumpkin seed meal and pumpkin peel in equal ratios, produced using an ultrasound-assisted method with glycerol-soy lecithin, demonstrated the highest tensile strength (1401 ± 5.4 kPa) and elongation at break (9.74 ± 0.46%). Such films could be applied in the packaging of foods such as bread, cakes, and candies. However, to allow these films to rival synthetic polymer films regarding mechanical and barrier qualities, it remains essential to establish a more economically sustainable commercial film-casting approach. PSPI and pumpkin peel pectin were mixed in different ratios and cast into edible composite films by standard methods, and the mechanical strength and barrier properties of the films were within acceptable ranges [106]. Preparing edible films using pumpkin processing by-products as raw materials not only reduced the production cost of food packaging but also achieved the dual goals of environmental protection and resource recycling, providing a theoretical basis and practical reference for the development of functional degradable packaging materials.

A novel biodegradable composite film was successfully prepared from pea starch and pumpkin seed protein [107]. Incorporating starch enhanced mechanical strength, hydrophobicity, and light transmittance of the composite. Notably, formulations with low starch content retained mechanical integrity after oil immersion, suggesting potential applications for oil-rich foods. The composite exhibited more tightly bound water molecules compared to pure PSPI films [107]. However, there was a corresponding reduction in elongation at break and a deterioration in water vapor and ultraviolet light barrier properties. In addition, composite pumpkin seed meal (PuOC) and bilayer pumpkin seed meal/maize protein films (PuOC/MZ) have been prepared by researchers [108]. Both PuOC/MZ and PuOC films exhibited a proficient oxygen barrier, markedly postponing the initiation of oxidative alterations in flaxseed oil. Molecular-level interfacial changes and process parameter optimization enable customized functionality in pumpkin seed protein composites. These advances make such materials viable candidates for functional packaging, especially in oil-based and oxygen-sensitive food preservation environments.

### 7.2. Meat Analogs

Plant-based meat, also known as alternative meat and artificial meat, is characterized by low saturated fat content, high protein levels, and the absence of cholesterol, providing many health benefits [109]. Owing to its balanced amino acid composition, favorable functional properties, and sustainability advantages, pumpkin seed protein exhibits diverse application potential in the field of meat analogs. Ebert et al. developed a hybrid dry-cured sausage using pumpkin seed protein, innovatively replacing 12.5% to 50% of pork with modified pumpkin seed protein [110]. During a 21-day maturation period, the dry-cured products containing 12.5% and 25% pumpkin seed protein showed drying characteristics, moisture distribution, and proximate composition highly similar to those of conventional meat products. Moreover, these products preserved the acidity characteristics and fundamental texture of conventional dry-cured sausages. In the processing of traditional sausages, PSPI also demonstrated practical value [111]. At an additive level of 1.5 g/100 g, the sensory score of the sausage reached 8.5 points. At this addition level, the product had a moisture, protein, and fat content of 51.16 g/100 g, 15.22 g/100 g, and 23.15 g/100 g, respectively, with a cooking loss of merely 6.98%. This enhanced the nutritious balance of the food while maintaining the quality attributes of conventional sausages.

Furthermore, PSPI may synergistically collaborate with mung bean protein isolate (MBPI) in the formulation of plant-based meat analogs when integrated with heat-induced gelation technology [112]. When MBPI and PSPI were blended at a ratio of 20:10, with the addition of 42% water and 7% maltodextrin (MC), a protein network was formed exhibiting great flexibility, and a moderately dense structure was established. Although the fibrous layered structure of animal meat had not yet been achieved, the synergistic effect between MBPI and MC significantly enhanced the product’s texture and viscoelasticity [112]. In addition, Choi et al. demonstrated that mixtures of SPI and pumpkin seed protein could be used to prepare high-moisture artificial meat [14]. The incorporation of pumpkin seed protein softened the gel texture while retaining the essential solid-like properties of meat, providing a new direction for the development of plant-based meat products [14]. In the future, approaches such as protein modification and optimization of blending systems can be used to further enhance its ability to mimic the texture and sensory attributes of meat, consequently propelling the advancement of meat analogs towards increased diversification and superior quality.

### 7.3. Carriers for Active Ingredients

Pumpkin seed protein is a favorable food formulation with potential as an encapsulation wall material for the efficient entrapment and delivery of active ingredients through various technological approaches. Čakarević et al. demonstrated the application of pumpkin seed protein in a beet juice encapsulation system, achieving encapsulation efficiencies of 92% and 75% for phenolic compounds via freeze-drying and spray-drying techniques, respectively [113]. Notably, the PSPI carrier exhibited slow-release properties in a simulated digestive environment. This offers the dual benefits of non-toxicity and nutrient fortification while maintaining the stability of bioactive compounds [113].

In a further study, biscuits containing three different levels of this encapsulant were used as a model system [99]. The utilization of PSPI as a carrier for bioactive compounds promotes its functional food applications and enhances the biological activity of the final food product. Concurrently, total phenolic and betaine content in the biscuits was increased, with an improved stability throughout storage [99]. Furthermore, PSPI served a protective function in preserving the structural integrity of astaxanthin under extreme conditions such as temperature variations and simulated digestion, attributed to its unique molecular conformation and compositional compatibility [114].

Oil-in-water emulsions are a class of emulsion systems that are of great importance in the food industry [115]. PSPI nanoparticles synthesized by the alcohol-induced anti-solvent approach have been effectively established as a vitamin E delivery system with remarkable ethanol tolerance [116]. The utilization of food-grade protein PSPI not only preserves the structural integrity of emulsions in complex processing environments but also offers protective functions and storage stability for oxidation-sensitive lipids. Moreover, PSPI has demonstrated promising applications in the field of tissue engineering. Kong et al. developed a core–shell structured microcarrier based on alginate, which utilized the cell-binding peptide properties of the PSPI shell layer to significantly enhance the adhesion efficiency of chicken myosatellite cells and primary porcine muscle cells [117]. This combination of nutritional functionality and interfacial affinity makes PSPI a highly promising 3D scaffold material for cell culture meat, opening up new avenues for bio-integration in future food manufacturing.

### 7.4. Protein Supplements

Pumpkin seeds are rich in protein and have been used as a partial replacement for conventional flour in various baked goods such as biscuits and bread. Slotted pumpkin seed flour is used to supplement protein in a variety of local foods. Studies have shown that wheat flour can be supplemented with up to 18% pumpkin seed protein concentrate or 22% pumpkin seed protein isolate without negatively affecting dough quality [118]. Furthermore, no negative effects on dough quality were observed. Bread supplemented with pumpkin seed protein exhibited 11–38% higher protein content and 55–80% greater levels of essential amino acids compared to control bread. As the protein level increased, dough stability time decreased, while dough softness improved. Galenko et al. further revealed that pumpkin seed protein concentrate has 5.2 times the total protein content of wheat flour and up to 2.3 times the fiber content [119]. Its addition not only increased the protein and unsaturated fatty acid content of the bread but also reduced the lignin level, thereby improving the digestibility and absorption of the bread. In the sensory evaluation, bread supplemented with 10% pumpkin seed protein concentrate achieved a higher sensory quality score, particularly in terms of flavor, texture, and overall acceptability. The remarkable sensory performance can be ascribed to the distinctive nutritional profile and structural attributes of pumpkin seed protein concentrate, which augmented the bread’s scent and improved its softness and chewiness [119]. This finding would contribute to promoting the expansion of pumpkin seed protein application in the field of functional baked foods.

In the field of gluten-free food, pumpkin seed protein has a wide range of potential applications. Tomić et al. devised a composite technique utilizing chickpea and pumpkin seed flours to effectively produce gluten-free biscuits [15]. This system avoided the use of conventional gluten ingredients while increasing biscuits’ protein, dietary fiber, phenolic content, and antioxidant capacity to varying degrees. Furthermore, pumpkin seed protein can be utilized to partially replace lentil flour in gluten-free lentil biscuits, increasing the protein ratio and modifying their physicochemical, functional, and sensory properties [120]. The substitution amplifies the Maillard reaction and yields a more intense baked scent, while the incorporation of pumpkin seed protein favorably influences antioxidant efficacy. In addition, a nutritionally enhanced gluten-free bread was developed by combining 13% pumpkin seed flour with 50% millet flour through recipe optimization [121]. Compared with traditional products, the protein and dietary fiber content increased by 50% and 30%, respectively, while the fat content significantly decreased by 70%. These innovative studies provide important technical approaches for the development of high-protein, low-allergenic functional baked products.

### 7.5. Functional Food Additives

Pumpkin seed proteins have substantial health advantages and have garnered increasing interest in recent years. As an important source of free tryptophan, the hydrolytic functionalization of pumpkin seeds has been shown to promote tryptophan release and enhance antioxidant activity [92], boosting the potential application in food ingredients and nutraceutical care products. Additionally, the antioxidant components present in pumpkin seed protein isolates effectively alleviated adverse effects induced by protein malnutrition and CCl_4_ toxicity [3].

Food-borne blood pressure-lowering peptides play a certain positive role in regulating blood pressure within the body. A novel tripeptide (IAF: Ile-Ala-Phe) with favorable bioavailability and an IC50 of 19.87 ± 0.50 μM was identified through the screening of new ACE inhibitory peptides derived from pumpkin seed proteins [76]. IAF can be regarded as a promising candidate for hypertension control and can serve as a raw material for the development of blood-pressure-reducing functional foods or drugs. Hydrolysates of transglutaminase cross-linked pumpkin seed meal protein have been reported as potential functional food additives [96]. When incorporated into a regular diet, this protein component serves as a natural antioxidant that is released during digestion and as a source of effective antihypertensive peptides. This demonstrates positive impacts on human health.

## 8. Challenges and Solutions

### 8.1. Limitations of Pumpkin Seed Protein in Digestibility

**Figure 4:**
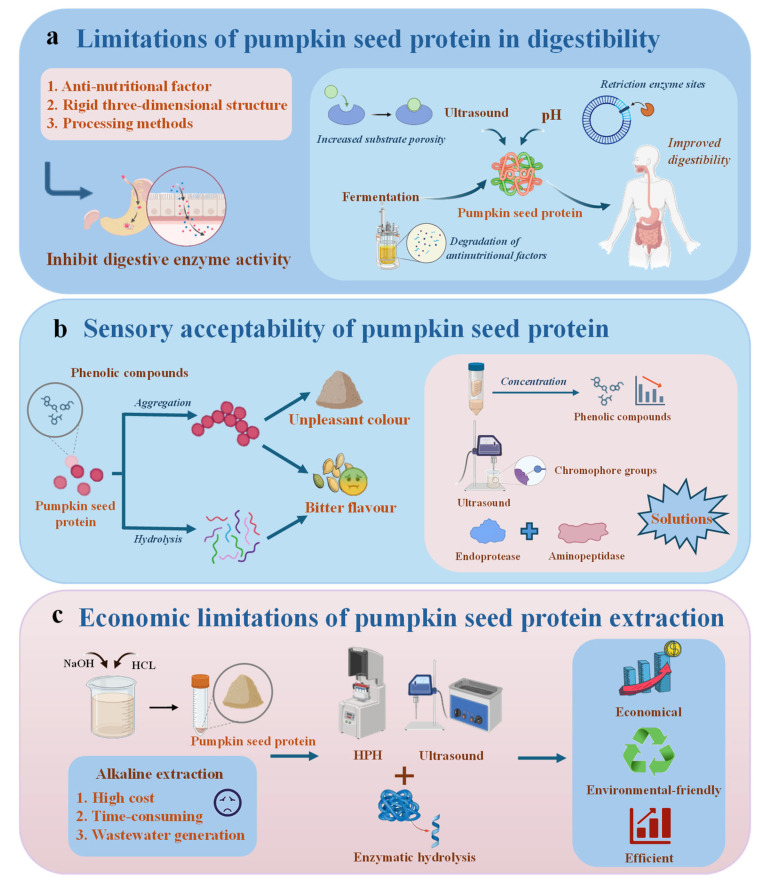
Challenges and solutions for pumpkin seed protein applications. (**a**) Limitations of pumpkin seed protein in digestibility. (**b**) Limitations of pumpkin seed protein in sensory acceptability. (**c**) Economic limitations of pumpkin seed protein extraction.

As a plant-based protein, pumpkin seed protein has low digestibility in its natural form [122]. Its in vitro digestibility is generally about 85%, which is closely related to its structural characteristics, the presence of anti-nutritional factors, and processing methods (Figure 4a). The dense 11S globulin forms a rigid three-dimensional structure through intramolecular disulfide bonds, which obscure the recognition sites of pepsin and trypsin. Simultaneously, anti-nutritional factors such as phytic acid and polyphenols directly inhibit digestive enzyme activity by forming complexes with the proteins [10,72]. The processing method chosen can also impact protein digestibility [9]. For example, the high temperatures during heat treatment induced protein cross-linking and amino acid racemization. Prolonged heat exposure even degraded essential amino acids and delayed their release.

Strategies for enhancing protein digestibility through structural modification involve multiple synergistic approaches. The change in pH can enhance the digestibility to 88.9% by modifying charge distribution and solubility. This induced protein unfolding to expose enzymatic cleavage sites and modified the composition of the secondary structure [80]. Non-thermal processing technologies demonstrated significant complementary effects. Combining the dual effects of heat treatment and alternating electromagnetic fields, microwave processing can reduce the activity of trypsin inhibitor by 65% while increasing the matrix porosity to facilitate enzyme penetration [123]. When combined with high-intensity ultrasound, pH modification of the proteins achieved 93.5% digestibility through simultaneous charge-mediated structural reorganization and cavitation-induced disruption of tertiary structure [67]. Giami et al. employed fermentation technology to treat pumpkin seed protein, resulting in an improved in vitro digestibility and a reduction in polyphenol and phytic acid concentration [124]. This biochemical approach not only restructured insoluble prolamins and glutelins to increase their susceptibility to pepsin but also enabled phytase-mediated conversion into inositol and phosphate, with simultaneous degradation of anti-nutritional compounds [125]. This mild biochemical treatment achieved a synergistic improvement in digestibility and bioavailability while maintaining the nutritional components.

### 8.2. Limitations of Pumpkin Seed Protein in Sensory Acceptability

Sensory acceptability presented a significant challenge in pumpkin seed protein applications. Proteins extracted from agricultural by-products typically exhibit undesirable flavor and color defects, a phenomenon that in pumpkin seed protein is intricately associated with the influence of phenolic chemicals within its matrix (Figure 4b). These substances not only act as anti-nutritional factors that reduce protein nutritional value but also form complexes with proteins via hydrophobic interactions and hydrogen bonds. On the one hand, they diminish sensory attributes, promote protein aggregation, and further aggravate flavor degradation. On the other hand, the phenolic chemicals cause increased protein color intensity, ultimately decreasing its visual acceptability [126]. Processing and concentration techniques may indirectly diminish phenolic content by reducing fiber, hence improving both nutritional and sensory attributes [72]. The application of HIU induced conformational changes in proteins through cavitation effects. This increased exposure of chromophore groups, enhancing brightness at higher ultrasonic power levels [50]. Simultaneously, HIU significantly altered the a* (red-green) and b* (yellow-blue) color parameters, resulting in greenish and pale yellow hues in the treated proteins [50]. This phenomenon offers a viable technical approach for color modulation in protein-based products.

Furthermore, enzymatic hydrolysis using proteases is widely regarded as an effective strategy for utilizing high-value bioresources, owing to its ability to enhance functionality and bioactivity. Nonetheless, when conventional endo-proteases were extensively utilized, they generally imparted significant bitterness to plant protein hydrolysates [127]. A synergistic strategy employing both endoproteases and aminopeptidases was developed, where the former cleaved polypeptide chains while the latter specifically removed N-terminal hydrophobic amino acids. This method improved hydrolysis efficiency and accomplished flavor alteration, offering an innovative strategy for bitterness reduction [66].

### 8.3. Economic Limitations of Pumpkin Seed Protein Extraction

In recent years, pumpkin seed meal has shown great potential for application in the food industry due to its high protein content (54%) and the specific functional properties of its plant proteins [6]. However, the existing extraction methods are difficult to effectively control the cost while considering the demand for commercial-scale production. Despite the operational simplicity and widespread use of alkaline extraction, its extended processing durations lead to significant energy requirements. Furthermore, the extensive use of alkaline solutions significantly exacerbates wastewater treatment challenges [36]. Consequently, it is essential to devise efficient, cost-effective, and environmentally sustainable extraction processes to extract pumpkin seed proteins on a commercial scale and to promote their utilization as food ingredients (Figure 4c).

High-pressure homogenisation and ultrasonic techniques demonstrate significant potential for optimizing alkaline extraction of pumpkin seed proteins in industrial applications. When integrated as supplementary steps into alkaline extraction protocols, these technologies not only reduced processing time and production costs but also enhanced protein recovery efficiency while minimizing nutrient loss [50,69]. In addition, enzymatic digestion protects against amino acid destruction due to the mild reaction conditions. The elevated expense of enzyme preparations and the limited protein output restrict its scalability [128]. Considering that enzymes and substrates can be physically or chemically modified to increase product yield under optimal conditions, there are many alternatives for controlling enzyme reactions. The amalgamation of ultrasonic pretreatment with enzymatic hydrolysis facilitates the process at diminished temperatures [78,89]. This approach decreased environmental impact and lowered production costs, while simultaneously accelerating hydrolysis rates and enhancing functional properties, thereby providing a practical solution for improved process economics.

## 9. Conclusions and Future Perspectives

As the demand for plant-based protein sources continues to grow, the nutritional value and potential applications of pumpkin seed proteins are attracting attention in the food industry. The development of value-added technology for pumpkin seed meal, an underutilized industrial byproduct, has significant economic and environmental benefits. Pumpkin seed proteins also possess certain functional properties, such as solubility, foaming, and emulsifying properties, as well as biological activities, such as antioxidant, antihypertensive, antidiabetic, antibacterial, and anticancer activities. Meanwhile, they have been shown various applications in the food field, including food packaging films, meat analogs, protein supplements, functional food additives, and as carriers of active ingredients. There are still some unresolved issues in the study of pumpkin seed proteins: (i) Current studies on the digestibility of pumpkin seed proteins mainly rely on in vitro digestive simulation models, which are unable to fully simulate the complex neuromodulation, immune response, and multi-organ synergistic effects in living organisms. It is necessary to combine animal experiments and organoid models to systematically investigate the absorption efficiency and metabolic mechanism of pumpkin seed proteins in authentic biological systems. (ii) Research into the functional properties of pumpkin seed proteins has mainly focused on their solubility and emulsification, but lacked an in-depth analysis of the conformational relationship between the multilevel structure of the proteins and macroscopic functions. Continuous research in extraction methods and modification techniques could enhance the yield and functionality of pumpkin seed proteins, thereby increasing their market competitiveness. (iii) The current knowledge of pumpkin seed protein about the allergenic fractions, epitope characterization, and cross-reactivity is severely lacking. A scientific allergy assessment model should be established to systematically characterize the sensitizing epitopes and their digestive stability. Sensitization reduction techniques, such as enzymatic modification and glycosylation treatment, should be explored to meet a wider range of nutritional and application requirements. Further studies on the above aspects should be conducted to promote the applications of pumpkin seed protein in the food industry.

## Figures and Tables

**Table 1:** Amino acid composition in different species of pumpkin seeds (g/100 g).

Amino Acid Composition	Pumpkin Seed Species					FAO/WHO	
	*C. maxima*	*C. moschata* var. *Kashi Harit*	*C. pepo* L.	*C. moschata*	*C. maxima Linn*	For Children	For Adult
Alanine	5.12	4.84	3.14	3.63 ± 0.05	5.07	-	-
Arginine	15.8	16.04	10.65	14.00 ± 1.00	9.06	-	-
Aspartic acid	9.56	7.12	7.22	11.94 ± 0.03	8.73	-	-
Cysteine	-	0.45	-	-	9.39	-	-
Glutamic acid	23.23	20.61	14.28	20.40 ± 0.10	3.37	-	-
Glycine	6.01	5.17	4.23	7.30 ± 0.70	5.63	-	-
Histidine	2.66	1.52	1.83	1.48 ± 0.06	2.16	1.90	1.60
Isoleucine	3.59	4.14	2.88	4.05 ± 0.03	3.51	2.80	1.30
Leucine	7.25	7.82	5.33	6.60 ± 0.03	7.24	6.60	1.90
Lysine	3.71	3.38	2.65	4.66 ± 0.02	2.78	5.80	1.60
Methionine	1.83	2.57	2.42	-	1.20	-	-
Phenylalanine	5.29	5.32	4.22	-	3.05	-	-
Proline	-	3.82	-	3.65 ± 0.01	2.32	-	-
Serine	5.85	4.43	3.97	4.90 ± 0.20	4.90	-	-
Threonine	3.04	2.19	1.88	1.39 ± 0.08	2.96	3.40	0.90
Tryptophan	1.10	2.10	-	-	-	1.10	0.50
Tyrosine	3.26	2.90	2.32	-	2.78	-	-
Valine	4.45	5.60	3.35	4.69 ± 0.03	2.40	3.50	1.30
References	[24]	[10]	[25]	[26]	[27]	[28]	[28]

*C.* represents *Cucurbita*; “-” represents the information missing or the work not reported.

**Table 2:** Amino acid scores (%) of pumpkin seed protein.

Amino Acid Composition	Preschool-Aged Children (FAO/WHO)				Adults (FAO/WHO)
	PSPI	WF	SF	AF	PSPI
Histidine	80.00	85.79	33.16	120.53	154.2 ± 1.9
Threonine	64.41	76.47	25.88	67.94	129.3 ± 1.0
Valine	160.00	133.43	64.57	156.57	125.6 ± 0.1
Methionine + Cysteine	120.80	102.80	442.00	130.80	119.9 ± 0.1
Isoleucine	147.86	91.43	74.29	147.50	142.6 ± 1.7
Leucine	118.48	88.79	84.70	119.09	109.2 ± 0.5
Phenylalanine + Tyrosine	130.48	82.22	69.84	147.46	217.5 ± 1.4
Lysine	58.28	88.97	31.38	54.66	86.5 ± 2.2
Tryptophan	190.90	105.45	143.64	206.36	211.1 ± 0.5
References	[10]	[10]	[10]	[10]	[9]

PSPI stands for Pumpkin seed protein isolate, WF stands for Water fraction, SF for Salt fraction, and AF for Alkali fraction.

**Table 3:** Extraction methods of pumpkin seed protein.

Extraction Methods	Extraction Conditions	Protein Content (%)	Advantages	Disadvantages	References
Alkaline extraction	At room temperature, pH 10.0 for 30 min, then the pH was adjusted to 5.0	94.3	Simple, low cost, and easy to extract	Long time, requires a large amount of buffer solution, and may also cause the loss of essential amino acids	[20]
	At room temperature, pH 11.0 for 30 min, then the pH was adjusted to 5.0	84.87			[29]
Ultrasonic-assisted extraction	20–25 kHz, 193.89 W, 19.08 min, 32 °C, pH 9.5	86.07	Simple operation and environmental friendliness	High cost and unsuitable for industrial scale	[30]
	456 W, 22 min, solid–liquid ratio 27 mg/L	81.86			[31]
	25 kHz, 60 min, 600 W/cm^2^, 50% amplitude	94.04 ± 0.77			[32]
	PEG 200-based DES concentration: 28% (*w*/*w*), solid–liquid ratio 28 mg/L, 43 °C, 140 W	93.95 ± 0.23			[33]
Enzymatic-assisted extraction	2% Alcalase, 2.4 L, pH 8.0, 50 °C	57.13 ± 0.65	Mild conditions, improved protein functional properties, and enhanced biological activities	High price and low protein extraction rate	[34]
	H1: Alcalase, 50 °C, enzyme to substrate ratio 0.5 mL/g. H2: Pepin, 37 °C, enzyme to substrate ratio 0.02 g/g	H1: 89.9 H2: 92.13			[29]

**Table 4:** Modification of pumpkin seed protein.

Modification Type	Modification Method	Structural Change	Effect	References
Enzymatic modification	Alcalase: 50 °C, 0.5 mL/g, 30 min Pepsin: 37 °C, 0.02 g/g, 90 min	Production of small peptides and free amino acids. Alterations in secondary and tertiary structures	Enhanced solubility, emulsification, surface hydrophobicity, and thermal stability	[20,60]
	Neutrase: 8000 U/g, 6 h		Improved solubility and antioxidant activity	[61]
	Pepsin:30 °C, 1%, 2 h		Enhanced emulsifying capacity	[62]
	Glutaminase: 45 °C, 1:100, 12 h		Improved solubility, foaming capacity, and OHC	[63]
	Alcalase: 60 min, 1:250 Flavourzyme: 120 min, 1:385		Improved antioxidant activity	[64]
	Aspergillus niger pepsin: 40 °C, 4.38 HUT/mg, 85 min		Improved antioxidant activity	[65]
	BAAP: 40 LAP, 12 h		Increased the small peptide content and antioxidant activity	[66]
Physical modification	Conventional thermal technology (87.8 °C, pH 8.0, 37 min)	Protein tertiary structure was disrupted, and hydrophobic groups were exposed; transitioned from natural conformation to semi-molten spheres	Enhanced FC (83.0%), WHC (1.90 g H_2_O/g), and OHC (0.90 g oil/g)	[9]
	Ultrasonic treatment (20 kHz, at 100 W, 300 W, or 500 W for 30 min)	Effectively changed the secondary and tertiary structure of the PSPI	Improved solubility and emulsifying properties	[50]
	150 W for 5 min		Improved foaming and emulsifying properties	[67]
	193.89 W for 19.08 min		Improved emulsifying properties and OHC	[30]
	40 kHz, 23.8 W/L, 25 °C, 120 min		Enhanced antioxidant capacity and improved color characteristics	[68]
	High-pressure homogenization (100 MPa)	Disruption of protein tertiary and quaternary structures by hydrogen bond cleavage	Solubility increased to 30.21 ± 0.93%; effectively improved the emulsification index of PSPI; FC increased to 24.58 ± 5.57%, but FS showed a decreasing trend	[69]
Chemical modification	pH-shifting treatment	At pH 2, 4, and 12, they possessed larger particle sizes and irregular morphology. At pH 6, 8, and 10, they exhibited a more uniform structure	Specific pH conditions maintained the thermal stability of the proteins and improved EAI. Extreme pH treatments more significantly improved surface activity and emulsion stability	[18,70]
	Acylation	Appearance of acetyl groups	Enhanced WHC, OHC, and emulsification properties	[71]
	Phosphorylation	Phosphate groups occurrence	Reduced levels of anti-nutritional factors and fiber content; significant increase in protein digestibility and essential amino acid content	[72]
	Fibrillation	The process of protofibrillation showed different structures	PSPI contained abundant hydrophobic protofibril-forming regions	[73]
	Complexed with bioactive components (EGCG, chlorogenic acid, gallic acid, pyrogallic acid (1,2,3-benzenetriol), apigenin, hydrogel network)	The secondary structure of the coupling was more regular and stable than that of PSPI	Enhanced antioxidant capacity of the protein, which also led to an increase in thermal stability; 66.07% increase in solubility, with a maximum activity of 421.91 m^2^/g, and free radical scavenging activities of 82.73% and 90.70% against DPPH and ABTS, respectively. Surface hydrophobicity. Improved antioxidant activity and release behavior in the gastrointestinal environment	[7,13,74,75,76]
Combined modification	pH + Ultrasonic treatment (20 kHz, 5 min)	Protein particles exhibited a narrower size distribution	Increased solubility, WHC, OHC, emulsification, and foaming properties; improved thermal stability and protein digestibility	[18]
	Heat + pH shifting	Destruction of the primary, secondary, and tertiary structures of proteins	PSPI emulsification capacity was significantly improved, with internal oil phase volume fraction up to 80%	[57]
	Ultrasonication (40 kHz, 450 W) +enzymatic treatment (Brauzyn and Neutrase: 25 °C, 60 min; Flavourzyme; 25 °C, 90 min)	The tertiary structure of the protein was disrupted, and the hydrophobic groups were exposed	Improved antioxidant properties and solubility	[77]
	Ultrasonication (40 kHz, 23.8 W/L) +enzymatic treatment (Flavourzyme: 25 °C, 120 min. Neutrase: 40 °C, 60 min)			[78]
	Microwave (500–900 W, 30–90 s) +enzymatic treatment (Trypsin: 1.5%, 105 min)	Secondary and tertiary structure disordered, hydrophobic groups exposed, surface hydrophobicity elevated	High iron chelating activity and DPPH radical scavenging capacity	[79]

## Data Availability

No new data were created or analyzed in this study. Data sharing is not applicable to this article.
